# Double Carbon Networks Reinforce the Thermal Storage and Thermal Transfer Properties of 1-Octadecanol Phase Change Materials

**DOI:** 10.3390/ma16227067

**Published:** 2023-11-07

**Authors:** Xiuli Wang, Qingmeng Wang, Xiaomin Cheng, Xiaolan Chen, Mingjun Bai

**Affiliations:** 1 School of Mechatronics and Intelligent Manufacturing, Huanggang Normal University, Huanggang 438000, China; wangxiuli@whut.edu.cn (X.W.); wangqingmeng@whut.edu.cn (Q.W.); chenxiaolan@hgnu.edu.cn (X.C.); 2 School of Materials Science and Engineering, Wuhan University of technology, Wuhan 430070, China; 3 Hubei Noble Vacuum Technology Co., Ltd., Huanggang 438000, China; baimingjun@hgnu.edu.cn

**Keywords:** double carbon network, 1-octadecanol, phase change thermal storage materials, heat transfer efficiency, photothermal conversion

## Abstract

Using thermal storage materials with excellent thermal properties in the energy utilization system enables efficient use of renewable energy sources. Organic phase change materials (PCMs) have the advantages of high heat storage density, no corrosion, and low cost, but low thermal conductivity and insufficient heat transfer capacity have always been the bottlenecks in their application. In this paper, melamine foam@ reduction graphene oxide (MF@rGO) and carbon foam@ reduction graphene oxide (CF@rGO) composite foams with double carbon networks were prepared by self-assembly method and further employed in 1-octadecinal (OD) PCMs. The microstructure, chemical composition, phase change behavior, thermal conductivity, and photothermal conversion performance of MF@rGO/OD and CF@rGO/OD were studied in detail using SEM, FTIR, Raman DSC, and LFA. The melting and solidification enthalpies of CF@rGO/OD composite PCMs were 208.3 J/g and 191.4 J/g, respectively, its thermal conductivity increased to 1.54 W/m·K, which is 6.42 times that of pure OD. The porous structure and high thermal conductivity of the double carbon network substantially enhance the efficiency of energy storage and release in composite PCMs. CF@rGO/OD composite PCMs have excellent heat storage performance and heat transfer capacity, and a wide range of application prospects in the fields of low-temperature solar heat storage, precision instrument temperature control, and intelligent buildings.

## 1. Introduction

With the prominent issues of energy shortage and environmental pollution, solar energy as a renewable and clean energy has been the focus of research. The use of phase change materials (PCMs) to realize the storage and reuse of intermittent energy such as solar energy has significant advantages [1,2]. Organic phase change materials have the characteristics of high heat storage density, wide sources, non-toxicity, and stable chemical properties, which are considered to be the most promising heat storage materials with broad application prospects in the fields of solar photothermal power generation, industrial waste heat recovery, electronic device heat dissipation, temperature control of textiles, and energy conservation of buildings [3,4]. The main limitations to the large-scale application of organic PCMs are common problems such as low thermal conductivity and easy leakage in the liquid state. Therefore, it is crucial to develop the new and high-performance shape-stable composite PCMs for the efficient utilization of solar energy and thermal energy, as well as the construction of sustainable energy-saving systems.

Carbon materials such as graphite, carbon nanotubes, carbon fibers, graphene, and carbon foam have superior thermal conductivity, low density, and good chemical stability [5,6,7]. The thermal conductivity and stability of heat storage materials can be significantly improved by adding carbon materials or combining them with PCMs to form carbon composite PCMs. Graphene-based composite PCMs have superior light absorption ability, high thermal conductivity and anti-liquid phase leakage, which have great potential for application in the field of solar energy conversion and storage. Akhiani et al. [8] added graphene oxide to palmitic acid to form a three-dimensional (3D) network structure through the self-assembly function of auxiliary oleamide. Graphene oxide could provide nucleation for the solidification of palmitic acid, which can retain 99.6% of the latent heat of phase transition of pure palmitic acid, the thermal conductivity can be increased by 1.5 times, and it has excellent thermal cycling. Cai et al. [9] used styrene-b-ethylene-co-butylene-b-styrene) (SEBS) as thickener, and reduced graphene oxide aerogel (rGOA) as multi-energy trapping agent and carrier, to prepare a multi-component and multi-functional composite PCMs. The interconnected rGOA skeleton serves as a continuous heat conduction path to accelerate heat transfer. However, the synthesis methods of graphene-based composite PCMs are usually complicated and costly, and rGOA is prone to agglomerate in the composite material, so the improvement of thermal conductivity is not obvious [10], greatly hindering large-scale preparation of practical application in thermal energy utilization technology. Reduced graphite oxide aerogel formed by graphene oxide (GO) self-assembly has the problems of high structural brittleness and poor mechanical properties, which greatly limits its large-scale preparation in thermal energy utilization technology [11]. Therefore, various skeletons or crosslinker molecules can be introduced by physical or chemical crosslinking to enhance the mechanical strength of rGOA [12]. Carbon foam has a 3D network structure, which is cheap and easy to obtain. The combination of carbon foam and PCMs can reduce the interfacial thermal resistance between the carrier and the core material, and greatly enhance the thermal conductivity of the composite materials, to realize the transformation from the microscopic scale to the macroscopic scale and to accelerate the energy conversion between the composite PCMs and external environment [13,14].

**Figure 1:**
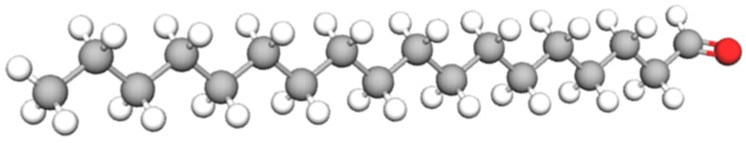
Molecular structure of OD.

1-Octadecinal (OD) PCMs with a phase change temperature of 57.0 °C has the advantages of high thermal enthalpy, stable physicochemical properties, and a wide range of sources. Figure 1 is the molecular structure formula of OD, which is composed of 18 C, 38 H, and one O, with -OH located at one end of the carbon chain. OD can play an important role in the field of low-temperature thermal energy storage, industrial waste heat recovery, precision instrument temperature control, and intelligent buildings. However, the low thermal conductivity (about 0.2 W/m·K) of OD and its susceptibility to leakage in liquid state seriously limit the practical application of OD. In order to solve the problems of OD and to give full play to the characteristics of graphene’s high thermal conductivity, this paper selects the commercially produced melamine foam (MF) as the skeleton, and prepares the carbon foam (CF) by simple high-temperature carbonization, and builds reduction graphene oxide (rGO) onto the interconnected fibers of MF and CF, so as to prepare melamine foam@reduction graphene oxide (MF@rGO) and carbon foam@reduction graphene oxide(CF@rGO) composite foam with a double carbon network, realizing the efficient preparation of high-performance composite foam. The composition, structure, porosity, and thermal conductivity of MF@rGO and CF@rGO composite foams were closely studied. The MF@rGO/OD and CF@rGO/OD composite PCMs were prepared by loading OD into the composite foam by vacuum impregnation method. The thermal conductivity, heat storage properties, shape stability, and photothermal conversion efficiency of the composite PCMs were researched, and the corresponding relationship between the microstructure and thermal properties of the composite PCMs were explored. Melamine foam and foam carbon were used as templates to construct graphene-foam carbon dual three-dimensional carbon network structures for composite PCMs by in situ deposition and hydrothermal reduction. The composite PCMs have excellent heat storage performance, high heat transfer capacity, and good mechanical properties, which realize the efficient, large-scale, and low-cost preparation of graphene-based foam-modified heat storage material. This work provides a theoretical basis for optimal design and performance prediction of thermal storage materials and systems.

## 2. Experimental

### 2.1. Materials

OD (CH_3_(CH_2_)_16_CH_2_OH) was purchased from China National Pharmaceutical Group Chemical Reagent Co., Ltd. (Shanghai, China); graphite oxide (GO) was obtained from China Nanjing Xianfeng Nano Material Technology Co., Ltd.; melamine foam was commercially supplied by China Zhengzhou Fengtai Nano Material Co., Ltd. The materials used in the sample preparation process were all raw materials without special treatment.

### 2.2. Preparation of Composite Foam

This article uses a simple high-temperature carbonization process to prepare composite foam. MF was cut into blocks of 50 × 20 × 15 mm and placed in a beaker. The MF was ultrasonically cleaned with deionized water and anhydrous ethanol successively, and the two reagents were used alternately two times, each time for 1 h. The cleaned MF was placed in a vacuum oven and dried at 60 °C. The pre-treated MF was placed in a tubular high-temperature resistance furnace with nitrogen as the protection gas. It was necessary to introduce nitrogen 20 min before heating to ensure a stable atmosphere in the furnace. The heating rate was 5 °C/min and the temperature was held at 600 °C for 1 h. CF was obtained after cooling.

**Figure 2:**
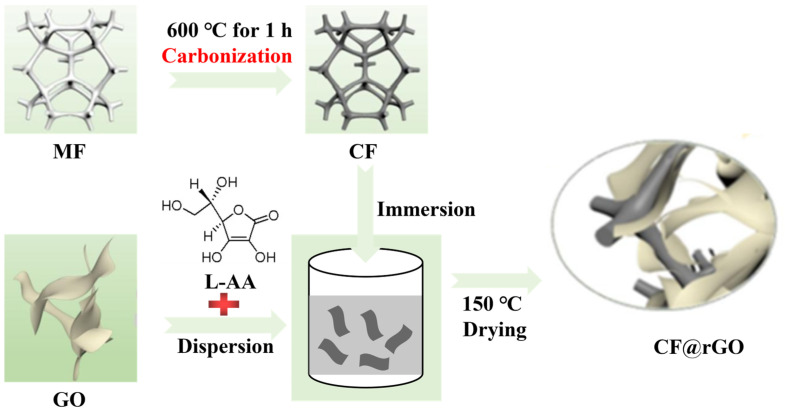
Preparation process of MF@rGO and CF@rGO composite foam.

MF has a continuous 3D network structure, excellent elasticity, and high compressive strength, but low thermal conductivity. The thermal conductivity of CF obtained from the carbonization of melamine foam is relatively high. The MF@rGO and CF@rGO composite foams with good strength, superior thermal conductivity, and a double carbon network were obtained by hydrothermal and chemical reduction reactions. MF contains -NH_2_ structurally, while GO contains oxygen-containing functional groups such as -OH and -COOH, which are amphiphilic. Therefore, GO, MF, and H_2_O have good affinity. MF@rGO and CF@rGO composite foam preparation steps were as follows: 50 mL GO solution (10 mg/mL) was put into a beaker and 0.1 g ascorbic acid was added with ultrasonic dispersion for 10 min; then, MF and CF were placed in the beaker and repeated pressure was applied to ensure the GO solution was fully in contact with the MF and CF. The beaker containing the sample was put into a vacuum oven at 80 °C to reduce GO to rGO under reducing agent and heating conditions. During the reduction process, rGO was self-assembled on the fiber connected with MF and CF, and MF@rGO and CF@rGO composite foam was obtained after being kept under vacuum for 12 h. The specific steps are illustrated in Figure 2. The composite foam was rinsed several times with deionized distilled water carefully to remove impurities in the sample, and then heated in an oven at 150 °C for 2 h to remove residual water.

### 2.3. Preparation of MF@rGO/OD and CF@rGO/OD Composite PCMs

MF@rGO/OD and CF@rGO/OD composite PCMs were prepared by the one-step vacuum impregnation method, which is simple and efficient. A sufficient amount of OD was put into a beaker, and the water was heated in a water bath at 90 °C until the OD was completely melted. MF@rGO and CF@rGO composite foams were placed into a beaker, and then kept in vacuum oven at 90 °C for 6 h. The blocks were taken out and cooled to obtain MF@rGO/OD and CF@rGO/OD composite PCMs.

### 2.4. Characterization

Field emission scanning electron microscopy (SEM, JSM-7500F, JEOL, Akishima, Japan) was utilized to observe the microstructure of the samples. A differential scanning calorimeter (DSC, NETZSCH, 200F3, Selb, Germany) was used to measure the phase transition temperature and latent heat of the sample, with a temperature range of 30–80 °C and a heating rate of 5 °C/min. Fourier transform infrared spectroscopy (FTIR, Thermo Nicolet USA) was utilized to analyze the chemical structure of the sample with a test range of 400–4000 cm^−1^. The spectroscopic data of the sample were calculated using a micro laser Raman spectrometer (Raman, INVIA, Britain) configured with a 633 nm laser with a CCD detector. An automatic surface area and pore analyzer (BET, ASAP 2460, Lafayette, LA, USA) obtained specific surface area and mesoporous structure data of samples by the nitrogen adsorption and desorption processes. The Discovery TGA (Waters, Milford, MA, USA) performed thermogravimetric analysis of samples in a nitrogen atmosphere at a temperature range of 20–500 °C at a heating rate of 10 °C/min. A Laser Flash Diffusivity Apparatus (LFA457, NETZSCH, Selb, Germany) collected the thermal diffusivity and thermal conductivity data of the sample at room temperature. The thermal diffusivity and thermal conductivity of each sample were measured three times to ensure the reliability of the experimental results.

## 3. Results and Discussion

### 3.1. Characteristics of MF@rGO, CF@rGO

#### 3.1.1. Morphology and Structure

**Figure 3:**
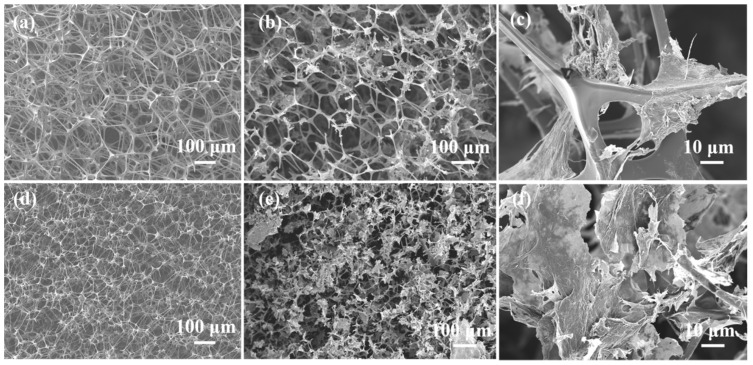
SEM images of (**a**) MF, (**b**,**c**) MF@rGO, (**d**) CF, (**e**,**f**) CF@rGO.

Figure 3 shows the microstructure of the MF, CF, MF@rGO, and CF@rGO composite foams. Commercial MF has a typical 3D network structure shown in Figure 3a. As shown in Figure 3b,c the MF@rGO composite foam maintained the network structure of the MF itself. rGO is constructed on the fibers of MF through reductive self-assembly, and this pore structure sufficiently supports the rGO in lamellar form, which increased the specific surface area of the composite foam and bound the PCMs within the pores. The rGO surface contained a large number of oxygen-containing functional groups, which gave a strong interfacial interaction between the GO sheet and MF. Figure 3d shows the 3D network porous structure of the CF skeleton with an aperture of about 50 μm. A large number of rGO sheets scattered in the 3D framework of CF perfectly formed semi-closed holes, and the graphene sheets were built on the CF skeleton in an unfolded shape, forming a double 3D network structure, as shown in Figure 3e,f. The multi-layer stacked RGO sheets in the gap of CF skeleton greatly improved the porosity of the composite foam. It improved the coating performance of OD and provided a heat channel for the subsequent heat transfer of the composite. CF@rGO composite foam not only had the 3D structure of CF and excellent mechanical properties, which restrained the PCMs to prevent the flow or leakage of the PCMs, but also took into account the thermal conductivity of rGO to provide a transmission channel for the heat, which greatly improved the efficiency of heat transfer and storage of the PCMs.

#### 3.1.2. Composition

**Figure 4:**
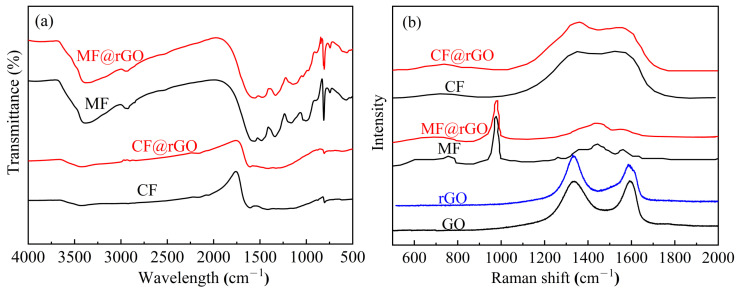
(**a**) FTIR and (**b**) Raman spectra of MF, CF, and composite foam.

Figure 4a displays the infrared spectra of MF, CF, MF@rGO, and CF@rGO composite foam. The main peaks of MF were 815, 1540, 2932, and 3380 cm^−1^, which are attributed to the bending vibration of the triazine ring, the stretching vibration of C=N, and the stretching vibration of -CH on the triazine ring [15,16]. Peaks in the 1200–1600 cm^−1^ range corresponded to C-N stretching vibrations of the S-triazine ring [17,18]. For MF@rGO, the peaks at 3293, 1479 and 1160 cm^−1^ showed a redshift, indicating that the amino group (N-H) of MF and the hydroxyl group (-OH) of rGO formed hydrogen bonds [19,20]. Meanwhile, both MF and MF@rGO had a sharp triazine ring deformation absorption peak at 811 cm ^−1^, indicating that there was still some melamine exposure in MF@rGO. Compared with the spectral curve of MF, it was found that the small peak of CF in the region of 1000–1600 cm^−1^ disappeared and was replaced by a wide peak. The peaks at 815 cm^−1^ disappeared, and the changes of these peaks indicated that the carbonization of MF was completed. The infrared profiles of CF@rGO were basically same as that of CF, mainly because the oxygen-containing functional groups were sharply reduced after the reduction of graphene oxide. The Raman spectra of MF@rGO and CF@rGO composite foams are given in Figure 4b. All samples exhibited two main peaks near 1340 cm^−1^ and 1596 cm^−1^ as well as D and G peaks. The D-peak was related to the disorder caused by structural defects in the material, while the G-band was caused by the E_2_g phonon scattering mode; i.e., the sp^2^ hybrid property of the carbon network. Therefore, the intensity ratio of I_D_/I_G_ is usually used to evaluate the degree of material disorder or the crystal size of graphite materials [21,22]. By calculating the I_D_/I_G_, it was found that the I_D_/I_G_ values of pure GO, rGO, MF@rGO, and CF@rGO composite foam were 1.23, 0.84, 1.12, and 1.37, respectively. The higher the disorder or the larger the sp^2^ structure, the higher the I_D_/I_G_. The Raman results further confirmed the reduction of GO in composite foams.

#### 3.1.3. Specific Surface Area and Porosity

**Figure 5:**
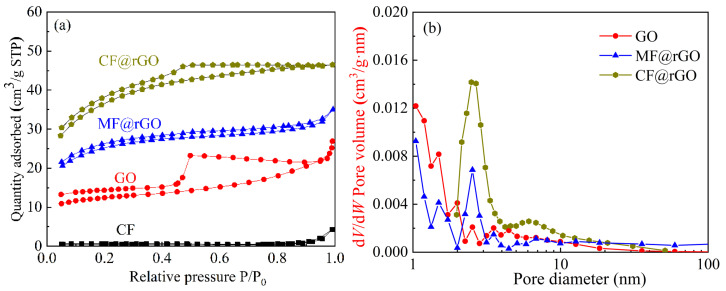
(**a**) N_2_ adsorption-desorption isotherms and (**b**) pore size distribution of CF, GO, MF@rGO, and CF@rGO.

Figure 5 shows the nitrogen adsorption and desorption isothermal curves and pore distribution curves of CF, GO, MF@rGO, and CF@rGO. The characteristic specific surface area (SBET), total pore volume (V_pore_), and average pore size of the samples were calculated using the Brunauer–Emmett–Teller equation, Barrett–Joyner–Halenda model, t-plot algorithm, and ask-plot algorithm [23]. The SBET of the MF was small, and there were no micropores and mesopores, so there were no MF specific surface area and pore-related data. As can be seen from Figure 5a, the SBET of CF was 1.7 m^2^/g, while the nitrogen adsorption-desorption isotherms of GO, MF@rGO, and CF@rGO all belonged to Type IV adsorption isotherms. The presence of adsorption hysteresis loops indicates that a large number of mesoporous pores were present in the sample. The adsorption–desorption curves of the three samples for N_2_ slowly increased in the low relative pressure region, indicating that a large number of micropores and mesopores existed within the GO, MF@rGO, and CF@rGO composite foams. Table 1 shows the specific surface area, pore volume, and aperture data of GO, MF@rGO, and CF@rGO. The specific surface area of GO was 46.0 m^2^/g, with a typical H3-type hysteresis loop. The functional groups on the surface of GO or rGO with two-dimensional structure and the graphite skeleton created strong interplane interaction, resulting in a greatly reduced surface area and only weak adsorption capacity [24]. The SBET of MF@rGO and CF@rGO were 82.6 m^2^/g and 120.9 m^2^/g, respectively. Both of them had pore distribution (D_pore_) in the range of 1~100 nm, with the dominant pore distribution being 2~10 nm and micropores < 2 nm. Porous carbons have long been studied due to their enhanced textural characteristics and customizable surface decoration. Bai et al. [25] prepared polyphenylene sulfide resin-derived S-doped porous carbons for efficient CO_2_ capture. The highest SBET properties were observed for one sample as 1227 m^2^/g. Lu et al. [26] fabricated disodium 2,6-naphthalene disulfonate (NDS)-derived self-S-doped porous carbon using a one-pot self-activating synthesis approach. The porous carbon exhibited excellent adsorption performance for CO_2_. In contrast, the MF@rGO and CF@rGO composite foams had simple structure, moderate porosity, and more easily adsorbed macromolecules of phase change materials. The MF and CF skeleton in the composite foam provided support points for rGO nanosheets to exist in lamellar form, thus greatly reducing the aggregation and overlap of rGO. The rGO nanosheets were connected to each other to form a large pore wall structure, while the large pore walls were close to each other and formed a medium-pore 3D fluffy rGO network structure with a larger specific surface area. The strong adsorption capacity and 3D structure of rGO are beneficial to the transfer of photogenerated electrons, and can improve the quantum efficiency so as to improve the photothermal conversion efficiency of composite phase change thermal storage materials [27]. The high specific surface area and large total pore volume of CF@rGO facilitated the high storage of OD, and induced capillary force, surface tension, and hydrogen bond force to ensure the stability of CF@rGO/OD composite PCMs.

### 3.2. Microstructure and Thermal Properties of MF@rGO/OD, CF@rGO/OD Composite PCMs

#### 3.2.1. Microstructure

**Figure 6:**
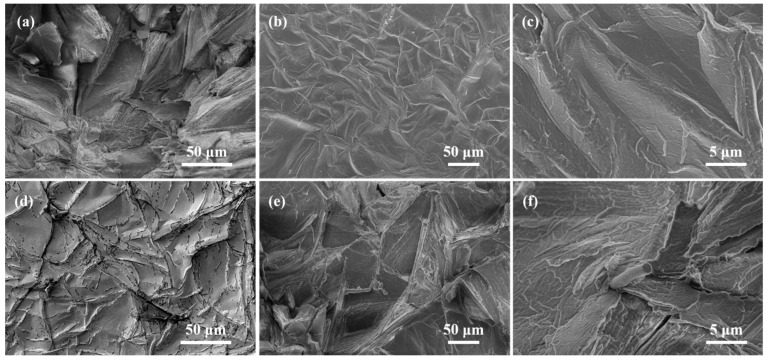
SEM images of (**a**) MF/OD, (**b**,**c**) MF@rGO/OD, (**d**) CF/OD, and (**e**,**f**) CF@rGO/OD composite PCMs.

Figure 6 shows the microstructure of MF/OD, CF/OD, MF@rGO/OD, and CF@rGO/OD composite PCMs. The overall darkness of the scanning image of MF/OD in Figure 6a is due to the low thermal conductivity of MF itself and the inability to improve the overall thermal conductivity when combined with OD. The pore structure of MF@rGO and CF@rGO was filled with OD, and no pore structure can be seen. The surface of MF@rGO/OD composite PCMs is wrinkled, and the rGO lamellae structure cannot be observed because OD filled in the 3D network structure of MF. Figure 6d clearly shows the CF covered with OD. The black spots scattered on the surface were caused by the melting of the sample due to the strong electron current during the shooting process, and the black spots are obviously clustered along the fiber structure. This phenomenon indicates that composite PCMs were subjected to the heat source, and due to the addition of composite foam, the heat was quickly transferred along the 3D carbon structure of the composite foam, thereby accelerating the melting of OD. In Figure 6e,f, OD was absorbed and filled inside the pores by the capillary action of the 3D network structure constructed by rGO, and the surface of the composite PCMs is flat; the CF skeleton can be clearly seen. MF@rGO and CF@rGO composite foams with high specific surface area and pore structure adsorbed OD firmly in their own pore structure by the capillary action and surface tension. The composite foam supported and covered OD well, effectively limiting the leakage and flow during the transformation of OD in the composite PCMs. In addition, the 3D skeleton of the composite foam interconnected in OD, which built a 3D network channel for heat transfer, thus greatly improving the heat storage and exothermic efficiency of the composite PCMs.

#### 3.2.2. Thermal Properties

**Figure 7:**
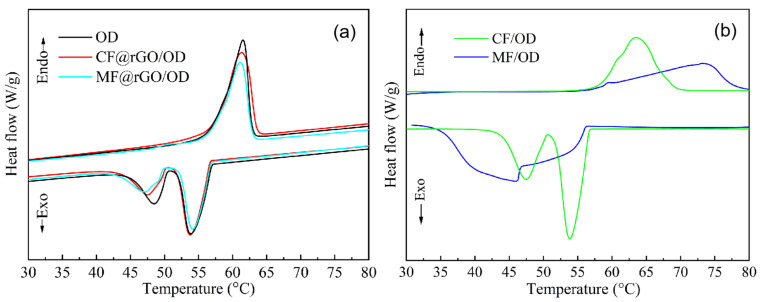
DSC curves of (**a**) OD, MF@rGO/OD, CF@rGO/OD, and (**b**) CF/OD, MF/OD composite PCMs.

The DSC curves and specific values of OD, MF@rGO/OD, CF@rGO/OD, MF/OD, and CF/OD composite PCMs are shown in Figure 7 and Table 2. In Figure 7a, the DSC curves of MF@rGO/OD and CF@rGO/OD composite PCMs basically coincide with that of the peak shape of pure OD, which all had one endothermic peak and two adjacent exothermic peaks, which indirectly indicates that the composite foam had no effect on the inherent heat storage and exothermic process of OD [28]. The DSC curves of composite PCMs in the melting process had an absorption peak, and the melting and solidification peaks of MF/OD were significantly wider, which is because the thermal conductivity of MF is low, and the thermal energy could not be transferred to other positions of the matrix quickly during the heating and cooling process. The melting temperature (T_m_) and solidification temperature (T_s_) of CF/OD were 56.9 °C and 56.8 °C, respectively. T_m_ and T_s_ of the composite PCMs were basically stable at an OD melting temperature of ±1 °C, indicating that the influence of CF on OD transformation temperature was relatively small. After the composite foam was combined with OD, it had no effect on the heat storage process of OD itself. Meanwhile, the composite foam only played an encapsulation role in the composite phase change thermal storage material, and had no contribution to the enthalpy of the composite PCMs. The phase change temperature and enthalpy values of MF@rGO/OD and CF@rGO/OD composite PCMs were only related to the loaded OD. As can be seen from Table 2, the T_m_ and T_s_ of MF@rGO/OD composite PCMs were 56.5 °C and 55.9 °C, and the T_m_ and T_s_ of CF@rGO/OD composite PCMs were 57.0 °C and 56.2 °C, and the OD structure was stable after being filled with composite foam. Lamellar graphene can increase the encapsulation of OD due to the existence of porous structure. There is a weak interaction between fatty alcohol molecules and the inner surface of the porous carrier material, thus causing a slight decrease in the phase transition temperature, and the improvement of thermal conductivity can accelerate the temperature response [29]. The melting enthalpies (ΔH_m_) of OD and MF@rGO/OD, CF@rGO/OD composite PCMs were 242.2 J/g, 192.7 J/g, and 208.3 J/g, respectively. The solidification enthalpies (ΔH_s_) were 210.1 J/g, 188.9 J/g, and 191.4 J/g, respectively, which accords with the requirement of thermal energy storage system for higher heat storage density of the heat storage medium. MF@rGO/OD and CF@rGO/OD composite PCMs had high phase change enthalpy and excellent heat storage capacity, which can provide an experimental basis for new carbon materials to improve the heat transfer and storage performance of organic solid–liquid phase variable heat storage materials.

#### 3.2.3. Thermal Conductivity

**Figure 8:**
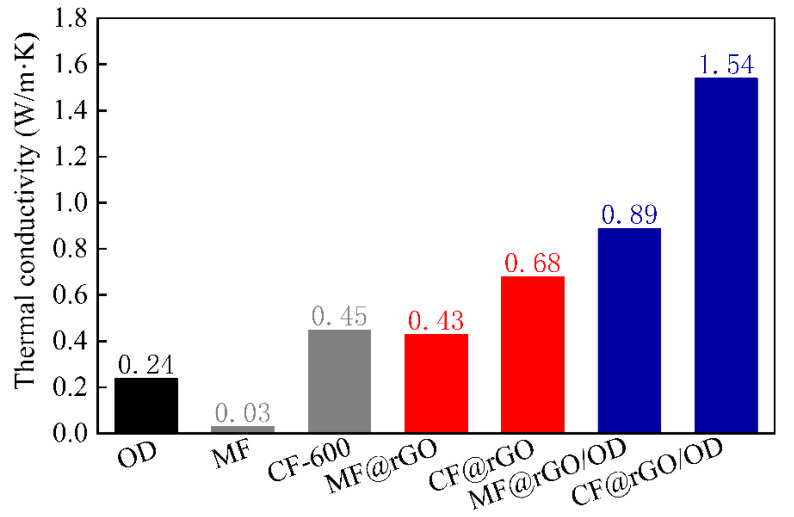
Thermal conductivity of OD, MF, CF, MF@rGO, CF@rGO, MF@rGO/OD, and CF@rGO/OD composite PCMs.

The thermal conductivity of OD, MF@rGO, and CF@rGO composite foam and MF@rGO/OD and CF@rGO/OD composite PCMs was tested, and the results are presented in Figure 8. The thermal conductivity of MF was only 0.03 W/m·K. After GO and MF were assembled to build a semi-closed 3D network hole structure, with the rGO layer providing a thermal conductivity path, the thermal conductivity of MF@rGO composite foam rose to 0.43 W/m·K, which is 14.33 times that of pure MF. The rGO greatly improved the thermal conductivity of composite foam. The thermal conductivity of MF after high temperature carbonization was 0.45 W/m·K, and that of CF@rGO composite foam was 0.68 W/m·K. The measured thermal conductivity of CF and CF@rGO composite foam was low. The main component of CF and composite foam is C, and the inherent thermal conductivity of C is very high in materials such as carbon nanotubes, C60, graphene, etc. During the thermal conductivity test, the composite foam was filled with air, as the thermal conductivity of air is very low, about 0.02 W/m·K, and the air in the pore structure greatly hindered the heat transfer between the CF and the composite foam [30]. The thermal conductivity of OD was 0.24 W/m·K, and that of MF@rGO/OD composite PCMs was 0.85 W/m·K, while that of CF@rGO/OD composite PCMs rose to 1.54 W/m·K, which was 6.42 times that of pure OD. The thermal conductivity of composite PCMs was greatly improved. When OD effectively filled in the porous structure of composite foam, the 3D pores had a large aspect ratio of continuous high thermal conductivity space vein to provide a 3D path for heat transfer, and improved the thermal conductivity of the composite PCMs.

#### 3.2.4. Shape Stability

**Figure 9:**
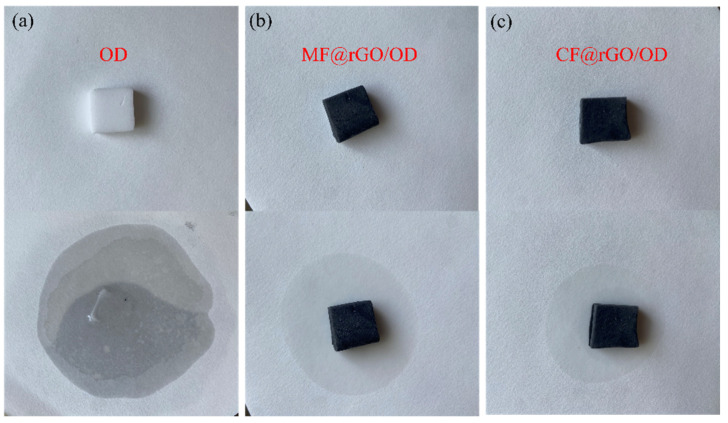
Photography of (**a**) OD, (**b**) MF@rGO/OD, and (**c**) CF@rGO/OD composite PCMs before and after thermal treatment at 90 °C for 3 min.

Since a dual carbon network can significantly enhance the thermal properties of OD PCMs, the shape stability and photothermal conversion of MF/rGO/OD and CF/rGO/OD composite PCMs were tested subsequently. The shape stability results of MF@rGO/OD and CF@rGO/OD composite PCMs are illustrated in Figure 9. OD, MF@rGO/OD, and CF@rGO/OD composite PCMs were placed on a hot plate at 90 °C with an adsorption paper underneath the sample to adsorb the molten OD promptly and show the adsorption circle. After 30 min, OD was completely melted and absorbed by the adsorption paper. OD could not maintain its original state without material encapsulation. MF@rGO/OD and MF@rGO/OD composite PCMs still maintained the original shape, with a small number of circles wet by OD at the bottom, mainly because the OD at the bottom of the composite PCMs was adsorbed due to the strong adsorption force of the adsorption paper after melting. The abundant network structure in MF@rGO and CF@rGO composite foam restricted OD to the interior and made it difficult for it to flow out. MF and CF served as supporting frames. The carbon network constructed by graphene on the frame had the function of a hollow shell, which further wrapped up the OD to form stable PCMs with a core-shell structure. MF and CF themselves have good mechanical properties, which effectively prevented the leakage of OD in the composite PCMs in the molten state, while the whole could withstand a certain degree of external force.

#### 3.2.5. Thermal Stability

**Figure 10:**
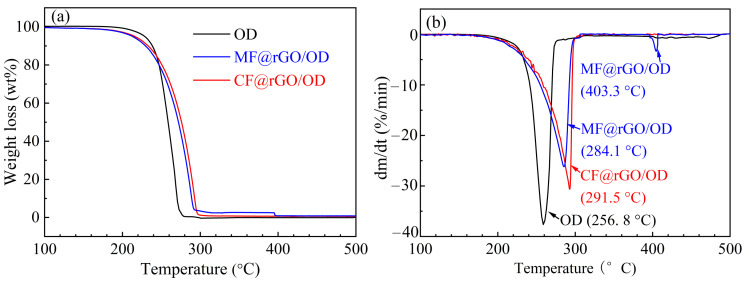
(**a**)TG and (**b**) DTG curves of OD, MF@rGO/OD, and CF@rGO/OD composite PCMs.

TG and DTG were important tests used to evaluate the thermal stability of the materials. Figure 10 shows the TG and DTG curves of OD, MF@rGO/OD, and CF@rGO/OD composite PCMs combined with the TG and DTG curves of OD. Combined with the TG and DTG curves of OD, it can be seen that OD only showed a single thermogravimetric process, which was the volatilization of the OD fat chain during the heating at 100 °C to 500 °C. The decomposition temperature of OD started at 151.2 °C, and the final end of decomposition temperature was 280.9 °C, with a mass loss rate of 100%. The initial decomposition temperatures of MF@rGO/OD and CF@rGO/OD composites were 148.0 °C and 145.5 °C, respectively, and the mass loss was obviously caused by the thermal decomposition of OD. The starting decomposition temperature and end decomposition temperature of MF@rGO/OD and CF@rGO/OD composite PCMs were higher than that of OD, which is mainly attributed to the amorphous existence of some phase change materials loaded in the composite foam, which increased the defects of OD and reduced its decomposition temperature [31]. As can be seen from the DTG curves of OD, MF@rGO/OD, and CF@rGO/OD composite PCMs in Figure 10b, the maximum weight loss rate corresponding to the temperature of OD was 256.8 °C. The maximum weight loss rates of MF@rGO/OD and CF@rGO/OD composite thermal storage materials were 284.1 °C and 291.5 °C, respectively. It is worth noting that the MF@rGO/OD composite PCMs showed a small weight loss at about 400 °C, mainly because the MF in the composite PCMs was carbonized and decomposed at 400 °C. MF@rGO and CF@rGO composite foam had improved ability to resist high-temperature decomposition after OD packaging, and to some extent widened the use temperature range of OD PCMs and improved its thermal stability, indicating that MF@rGO/OD and CF@rGO/OD composite PCMs have better thermal stability in the field of low-temperature phase change energy storage.

The important parameters for thermal energy storage materials are phase change temperature, thermal enthalpy, and thermal conductivity. Table 3 lists the thermal properties of composite PCMs in related literature. Researchers analyzed the impact of various porous carbon materials on the performance improvement of phase change materials. Zhou et al. [32] prepared hierarchical porous carbon surface-decorated graphitic carbon foam (SGF), which served as an encapsulated skeleton for stearic acid (SA). The SA/SGF exhibited good thermal stability after 200 thermal cycles. Wu et al. [33] introduced a novel shape-stabilized SA–CF composite PCM with a continuous dual-scale interpenetrating network structure. The composite presented a thermal conductivity of 1.30 W/m·K and displayed excellent thermal cycle stability. Moreover, the methods of this work were simple and low-cost. CF@rGO/OD composite PCMs have high thermal enthalpy (208.3 J/g) and high thermal conductivity (1.54 W/m·K). Consequently, the composite has great potential in solar storage and waste heat recycling.

#### 3.2.6. Photothermal Conversion

**Figure 11:**
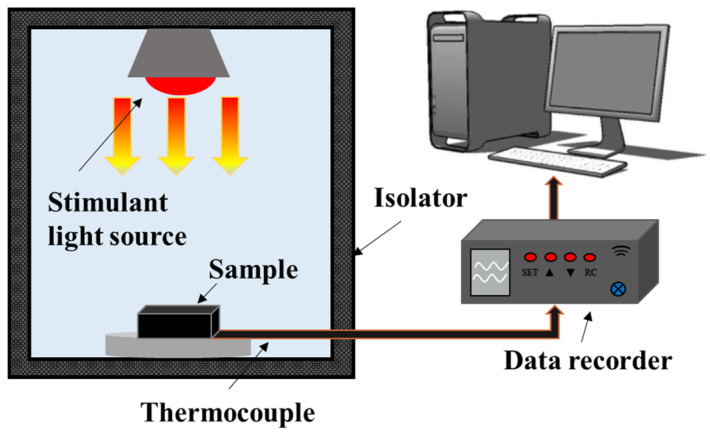
Apparatus for testing the photothermal conversion performance.

**Figure 12:**
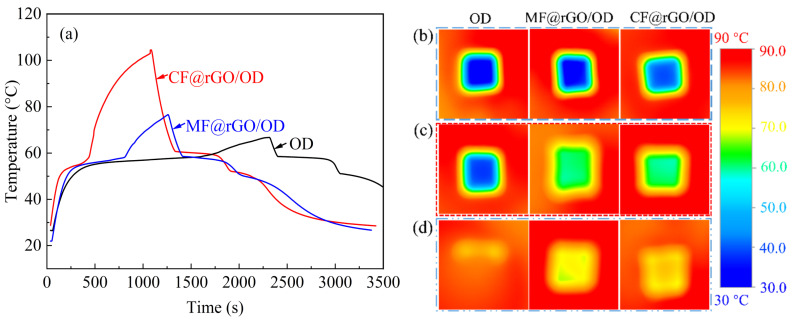
(**a**) Photothermal conversion curves and IR thermal images at (**b**) 0 s, (**c**) 30 s, and (**d**) 100 s of OD, MF@rGO/OD, and CF@rGO/OD composite PCMs.

The conversion and storage of photothermal energy are crucial in solar energy collection systems, and it is difficult for conventional media to balance high photothermal conversion, high thermal conductivity, and high thermal energy storage. Graphene nanosheets have excellent photon capture characteristics and high thermal conductivity, making MF@rGO and CF@rGO composite foams an ideal photothermal conversion substrate for solar energy capture and application [37]. In order to test the photothermal conversion performance of composite PCMs, the photothermal conversion testing system was built independently as shown in Figure 11. The main components of the device consisted of an insulation system (during the testing process, the vast majority of thermal radiation is isolated by tin foil), a hernia lamp (CEL-NP2000, China Education Aurora), T-type thermocouples for testing temperature, and a multi-channel data collection system. The sample were placed in an insulated box under simulated sunlight irradiation, and a data logger automatically recorded the temperature changes of the samples during the testing process. Figure 12a shows the photothermal conversion characteristics of MF@rGO/OD and CF@rGO/OD composite PCMs with and without sunlight irradiation conditions. Under the radiation conditions, the temperature rise was very slow due to the poor temperature absorption of pure OD. In contrast, the MF@rGO/OD and CF@rGO/OD composite PCMs warmed up rapidly under sunlight irradiation. All three showed a heat-absorbing plateau around 57 °C, which corresponded to the process of solar energy storage by the matrix OD through its own phase transition. It is worth noting that the endothermic platform of CF@rGO/OD composite PCMs was very short, while the platform of pure OD lasted about 1200 s, which fully demonstrates that the composite foam enabled the composite PCMs to realize rapid heat charge and discharge. The time required for the OD, MF@rGO/OD, and CF@rGO/OD composite PCMs to rise from 30 °C to 60 °C was 1684 s, 752 s, and 407 s, which further demonstrates that the addition of composite foam improved the thermal properties of OD. The rGO of MF@rGO and CF@rGO composite foam could be used as a medium to absorb solar energy and convert it into heat, and the heat generated was transferred to OD PCMs through the thermal conductivity channel in the composite foam structure. When the radiant sunlight was turned off, the composite PCMs began to rapidly cool until another platform appeared, and OD released the stored heat energy through solidification. In order to investigate the photothermal conversion capability of the prepared composite PCMs, the photothermal conversion efficiency (*η*) of the samples was calculated by Equation (1).

(1) $$\eta =\frac{m\cdot △H}{P\cdot S\left(t_{0}-t_{e}\right)}$$

where *m* is the quality of the test sample, Δ*H* is the latent heat of phase transition of the sample, and the data can be obtained from DSC. *P* and *S* are the solar radiation intensity (200 mW/cm^2^) and the area of the sample that can receive radiation; *t*_0_ and *t_e_* are the beginning and end times of the phase transition.

The conversion rates of OD, MF@rGO/OD, and CF@rGO/OD composites were 38.6%, 70.2%, and 90.9%, respectively. Considering the similar weight, size, and enthalpy of the samples, the reason for the improved photothermal storage efficiency of the composite material was that the porosity of CF and the high specific surface area of rGO greatly increased the adsorption and contact with OD. The rGO skeleton in the composite foam provided a good heat transfer channel for the adsorbed OD, accelerating the heat transfer and heat storage efficiency of the corresponding PCMs, which led to the elevation of the heating rate of the composite PCMs, leading to a shorter phase change cycle [38]. MF@rGO/OD and CF@rGO/OD composite PCMs have excellent solar thermal storage efficiency and can be confirmed as the optimal medium for practical solar energy storage applications.

The thermal conductivity of MF/rGO/OD and CF/rGO/OD composite phase change thermal storage materials was visualized and compared by evaluating their temperature response during heating and cooling. An infrared thermal camera was used to record the changes of surface temperature of the composite PCMs when they were placed on the hot and cold platforms, as shown in Figure 12b. During the heating process, the temperature rise rate of CF@rGO/OD composite PCMs was faster than that of MF@rGO/OD composite PCMs, until OD melted, resulting in a constant temperature platform. After the melting transformation was completed, the temperature of CF@rGO/OD composite PCMs began to rise rapidly. As expected, similar results were observed during the cooling process, and the CF@rGO/OD composite PCMs exhibited better heat transfer rate. In addition, the more uniform color on the surface of the CF/rGO/OD composite PCMs confirmed the uniform heat distribution, which can be attributed to the dense and uniform injection of OD into the composite foam. The higher heating and cooling rates of CF@rGO/OD composite PCMs indicate faster heat transfer characteristics.

## 4. Conclusions

MF and CF were selected as the skeleton, and GO was induced to self-assemble on the surface of skeleton to build a 3D rGO network structure through the reducing agent and heating treatment, which effectively improved the interfacial bonding strength between graphene and the foam matrix, and preliminarily realized the highly efficient and low-cost preparation of graphene-based composite foams. MF/OD, CF/OD, MF@rGO/OD, and CF@rGO/OD composite PCMs were prepared by the vacuum adsorption method. The effects of MF@rGO and CF@rGO composite foams on the encapsulation effect, microstructure, chemical composition, phase change behavior, thermal conductivity, and photothermal conversion performance of OD were systematically studied. The relationship between composition, microstructure, and properties of MF@rGO/OD and CF@rGO/OD composite PCMs were established. The specific results are as follows:

(1) For MF@rGO and CF@rGO composite foams, rGO sheets were uniformly built in the 3D framework of MF and CF to form a dual 3D network structure interspersed with each other. The specific surface areas of MF@rGO and CF@rGO composite foams were 82.6 m^2^/g and 120.9 m^2^/g, respectively. The high specific surface area and large total pore volume were conducive to the high storage of OD organic molecules.

(2) The melting enthalpies of MF@rGO/OD and CF@rGO/OD composite PCMs were 192.7 J/g and 208.3 J/g, and the solidification latent heat values were 188.9 J/g and 191.4 J/g, respectively, which meet the requirements of thermal energy storage medium for high energy storage density. The thermal conductivity of MF@rGO/OD and CF@rGO/OD composite PCMs was 0.85 W/m·K and 1.54 W/m·K, respectively. The porous structure and high thermal conductivity of the composite foam significantly improved the energy storage and release efficiency of the composite PCMs.

(3) The rich network framework structure of composite foams provided huge surface tension and capillary force to support the shape stability of OD before and after phase transition. Compared with OD, the maximum weight loss rate of MF@rGO/OD and CF@rGO/OD composite PCMs increased by 27.3 °C and 34.7 °C, respectively. Both MF@rGO/OD and CF@rGO/OD composite PCMs had good thermal stability in low-temperature phase change energy storage.

(4) The rGO skeleton in the composite foam provided a suitable heat transfer channel for the adsorbed OD, accelerating the heat transfer and storage efficiency of the corresponding PCMs. Composite PCMs have good application prospects in the fields of heat storage and heat management.

## Figures and Tables

**Table 1:** Porous structure parameters of CF, GO, MF@rGO, and CF@rGO.

Samples	SBET (m^2^/g)	V_pore_ (cm^3^/g)	D_pore_ (nm)
CF	1.7	—	—
GO	46.0	0.04	3.55
MF@rGO	82.6	0.09	8.17
CF@rGO	120.9	0.08	14.28

**Table 2:** Phase change temperature and thermal enthalpies of OD, MF@rGO/OD, and CF@rGO/OD composite PCMs.

Samples	Melting		Solidifying	
	T_m_ (°C)	ΔH_m_ (J/g)	T_s_ (°C)	ΔH_s_ (J/g)
OD	57.7	242.2	56.7	210.1
MF/OD	56.9	181.7	56.6	177.9
CF/OD	56.9	239.0	56.8	201.2
MF@rGO/OD	56.5	192.7	55.9	188.9
CF@rGO/OD	57.0	208.3	56.2	191.4

**Table 3:** Comparison of thermal properties of different composite PCMs in the literature.

Filler	Matrix	T_m_ (°C)	ΔH_m_ (J/g)	Thermal Conductivity (W/m·K)	Ref.
Surface-decorated graphitic carbon foam	stearic acid	71.3	167.5	3.25	[32]
Dual-scale pore carbon foam	stearic acid	70.5	192.8	1.30	[33]
CNT/expanded perlite	paraffin	43.7	95.9	0.50	[34]
Nano-TiO_2_/carbon nanofiber	OD	57.6	209.1	0.43	[35]
NiO@CF	OD	56.5	185.3	1.12	[36]
CF@rGO	OD	56.5	208.3	1.54	This work

## Data Availability

Data are contained within the article.

## Abbreviation

Nomenclature		Abbreviate	
T_m_	melting temperature (°C)	PCMs	phase change materials
T_s_	solidification temperature (°C)	OD	1-octadecinal
ΔH_m_	melting enthalpies (J/g)	MF	melamine foam
ΔH_s_	solidification enthalpies (J/g)	CF	carbon foam
V_pore_	pore volume (cm^3^/g)	3D	three-dimensional
D_pore_	pore distribution (nm)	SEBS	styrene-b-ethylene-co-butylene-b-styrene
η	photothermal conversion efficiency	rGO	reduced graphene oxide
P	solar radiation intensity (mW/cm^2^)	rGOA	reduced graphene oxide aerogel
S	area of the sample	SBET	specific surface area
m	quality (g)		
t_0_	beginning time of the phase transition (s)		
t_e_	end time of the phase transition (s)		
